# Focusing a mobile science learning process: difference in activity participation

**DOI:** 10.1186/s41039-016-0040-6

**Published:** 2016-07-30

**Authors:** Daner Sun, Chee-Kit Looi

**Affiliations:** 1 Department of Mathematics and Information Technology, The Hong Kong Institute of Education, Hong Kong, SAR China; 2 National Institute of Education, Nanyang Technological University, Singapore, Singapore

**Keywords:** Mobile technology, Science curriculum, Activity participation

## Abstract

The research on mobile learning in science lacks in-depth investigation of the learning process. In this paper, we describe the implementation of a mobile technology-supported science curriculum developed by design-based approach. The long-term data collection and trace of learning process enable the exploration of students’ participation and identifying potential factors in mobile learning. Employing mixed research methods, the study presents the differences of students’ engagement in mobile activity. It was found out that the participation of students in doing the mobile activities varied regarding the types of mobile tools, topics, class levels, and teacher feedback. The findings unfolded the factors affecting student activity participation behavior in mobile science learning and the problems encountered by the mobile science curriculum implementation. The results could potentially inform curriculum design and implementation supported by mobile technology, as well as support professional development of science teachers.

## Introduction

With the advance of the Information and Communication Technology (ICT), mobile devices (e.g., smartphones, tablets, handheld science sensors) have been absorbed into the fabric of our daily lives rapidly (Merchant 2012). In education, the ways of integrating various mobile technologies into the curriculum have been extended and elaborated in some subject domains. Science has been one of the most prominent subjects in which learning has been enhanced by appropriating mobile technologies. Relevant studies have demonstrated that features of mobile technologies could better serve for the science learning taking place in informal learning contexts (Song et al. 2012; Looi, et al. 2014b; Sharples et al. 2014).

In science education, research on technological design, pedagogical development, and implementation and evaluation of mobile devices enabled learning has been accumulating, yet challenges remain in supporting teacher enactment and documenting evidence of student learning process in the context of mobile learning. The researchers and educators have recognized the importance of getting insights into the mobile learning in the informal contexts, but challenges on the curriculum design and implementation, as well as the assessment of students’ learning process exist. Literature review has indicated that the depth of investigation of learning in informal contexts is much less than researchers’ efforts on formal learning (i.e., classroom). Mortensen and Smart (2007) points out that although there is a growing effort to create partnerships between schools and informal learning settings, documentation of such projects is limited. And most of relevant studies generally reported such projects as examples of “best practice” with few discussions of challenges before or during implementation. These are the issues encountered by research on mobile learning which locates more in informal learning contexts. Thus, the current issues promote this study which focuses more on capturing evidence from students’ mobile learning activities in the informal contexts, with the major purpose of exploring students’ learning process via examining the participation in activities. In this paper, a science curriculum supported by mobile technology is introduced. The participation of students’ doing activities with different mobile tools is analyzed related to the class levels, topics, and teacher feedback. The relations between students’ participation and teacher feedback for mobile learning are further discussed. The findings and discussions will inform the curriculum design and implementation deploying mobile technology in science education and the teacher professional development of science learning in the informal contexts.

## Background

### Formal learning and learning in informal contexts

In science education, learning outside of formal institutions is certain to be of growing importance in relation to the formal school curriculum (Gokpinar and Reiss 2016; Wellington 1990). Morag and Tal (2012) believed that regardless of how they were defined, all out-of-school learning environments had a variety of cognitive, affective, social, and behavioral effects that could make a significant contribution to learning. Hofstein and Rosenfeld (1996) contended that “it would be useful if science educators would consciously utilize a wide range of out-of-school environments which foster science learning.” They preferred to adopt the “hybrid” view (rather than the dichotomy view) that informal learning experiences can occur in formal learning environments (e.g., schools) as well as in informal learning environments (e.g., museums, zoos). They suggested that future research in science education should focus on how to effectively blend informal and formal learning experiences in order to significantly enhance the learning of science. Bell and others (2009) shared the same viewpoint that informal learning contexts should be seen as complementary to formal schooling rather than as in competition with it. Their report responded to the need for greater coherence and integration of informal environments and K-12 functions and classrooms, and the report urged a careful analysis of the goals and objectives of science learning in informal environments. Mortensen and Smart (2007) pointed out that although there was a growing effort to create partnerships between schools and informal learning settings, documentation of such projects is limited and generally reported as examples of “best practices” with little discussion of challenges before or during implementation.

In their opinions, an informal education venue could be a valuable resource that reinforces classroom pedagogy. Therefore, new questions raised about how and what aspects of formal learning and learning in informal contexts should be connected and integrated into the schooling system.

### Mobile learning for science education

With mobile technology, the science learning environment can be mobile and moves with the students to the field site, to the laboratory and beyond (Martin & Ertzberger 2013; Zydney & Warner 2016). According to Hwang and Tsai (2011), despite the multiple definitions of mobile learning, each focusing on a different aspect, they shared the same idea, that was, the mobile device played an important role in the learning activities no matter whether the activities were conducted in the field or in the classroom. Mike Sharples et al. (2009) mentioned that mobile learning offered new ways to extend education outside the classroom, into the conversations and interactions of everyday life. The use of mobile devices blurs the distinction between formal and non-formal learning. The extension of the learning environment enables students to investigate more science phenomena in real life and to demonstrate principles and scientific knowledge in different contexts other than the laboratory or the classroom (Shih et al. 2010). Furthermore, social networking opens up opportunities for students to do socially mediated knowledge building associated with learning science by doing science at anytime and anywhere.

Recently, the most frequently discussed issues are the missing aspects of how students think, discuss, and reason when they interact with the informal learning environments. Thus, more fine-grained analysis is needed to better understand the processes by which mobile technology merges into the learner’s daily life and to look into the ways in which technology is used and integrated in students’ daily life (Rogers & Price 2008).

### Existing studies of mobile learning in science

Rogers and Price (2008) incorporated the use of mobile tools in science guided by the collaborative inquiry principle in students’ field trips. Results showed that the tools helped students engage more in the discussion, interpretation, sharing of, and reflection upon their inquiry. Song et al. (2012) proposed a goal-based approach to design a mobile curriculum to guide students’ personalized inquiry learning. The approach proved effective in terms of developing students’ scientific knowledge and self-directed learning skills. Ahmed and Parsons (2013) developed a mobile learning system called ThinknLearn for supporting students’ abductive science inquiry throughout the process of exploration, examination, selection, and explanation. The findings indicated that students enhanced their skills on generating hypotheses and critical thinking. These studies collectively point out that mobile technologies can play active and mediate roles in science education either in and out of the classroom, and once the appropriate pedagogical approaches (e.g., inquiry-based principles) are incorporated, there is great potential for the improvement of students’ knowledge, skills, competences, and attitudes toward science in and out of the classroom.

However, as Sharples mentioned early on, an instructional design theory for mobile learning has not been fully articulated (Sharples et al. 2009). In reviewing the published reports, while most were about creating a learning environment for leveraging the affordances of mobile technologies, the learning experiences they supported were short-term and practice-oriented rather than theory purpose in nature (Sun et al. 2015). There is few research that takes a holistic approach to defining and realizing sustainable learning with mobile technologies via immersing it into the national standard science curriculum for sustainable and scalable implementation and improvements. Also, little effort has been made to trace the trajectory of the transformation of teacher and student behaviors achieved through long-term innovation. More evidence were expected to collect to inform the relevant studies on mobile technology use outside of the classroom.

## Research questions and purposes

This study was conducted to answer the following research questions:

- How to capture students’ activity performance in the informal contexts?
- What are the differences of the participation when students doing various mobile leaning activities?
- What is the relationship between teacher feedback and students’ activity participation in mobile learning activities?

With answering the above research questions, our research aims to present long-term efforts of mobile science curriculum implementation, with focusing on introducing the design features of the curriculum, the analysis of students’ participation performance in the mobile activities, and finally exposing the factors affecting students’ behavior in mobile learning context.

## Methods

### Features of the M5ESC

The curriculum is named Mobilized 5E Science Curriculum (M5ESC). The learning design is facilitated by the 5E instructional model which has been frequently integrated with the science instruction in primary and secondary levels. The 5E instructional model refers to the doing of science learning followed by five inquiry phases: engage, explore, explain, elaborate, and evaluate (Bybee et al. 2006). The 5E inquiry allows students and teachers to experience common activities, to use and build on prior knowledge and experience, to construct meaning, and to continually assess their understanding of a scientific concept. When integrated with the use of mobile technology, the 5E inquiry learning goes beyond the walls of the classroom and students’ interaction with the outsides become more frequent. For example, with mobile technologies, students will observe the science phenomena and collect science data at home or outsides at the stage of Explore, and they will post explanations via mobile tool at the stage of Explain and then with the guide of the teacher, they will elaborate their ideas when come back to the classroom at the stage of Elaborate. With flexible integration of the mobile tools with the inquiry phases, the learning effectiveness of mobile technologies for science inquiry will better delivered.

**Fig. 1:**
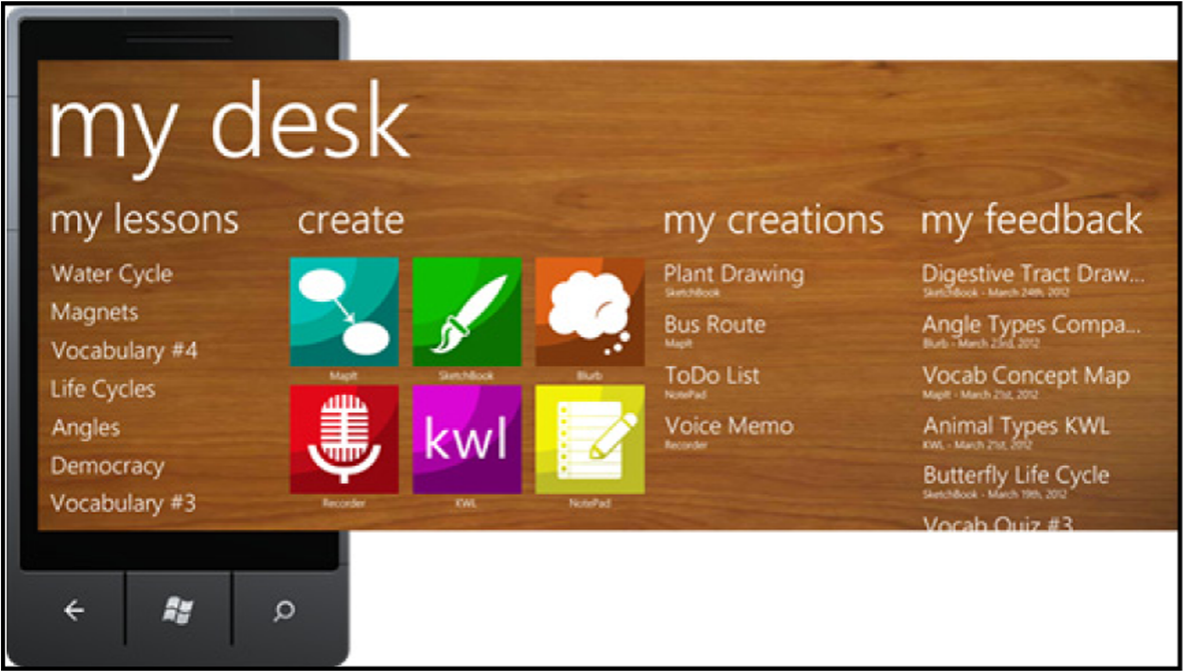
MyDesk student version

**Fig. 2:**
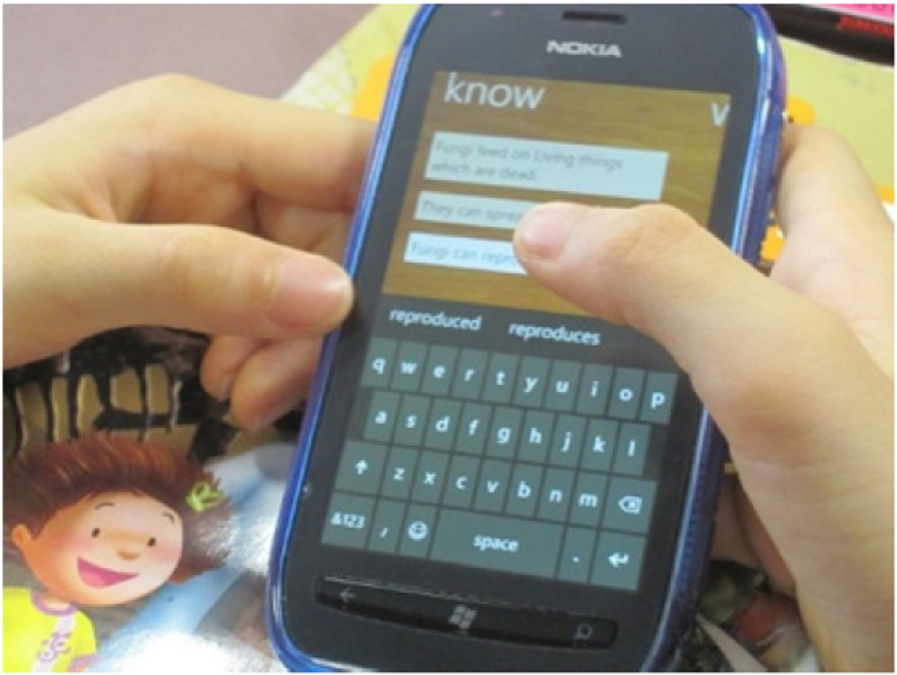
Student doing KWL activity

In the M5ESC, a learning management system MyDesk was developed for supporting the teachers to design the mobile activities and manage students’ work generated in the system. The MyDesk system consists of two versions. Student version is installed in the windows smartphone for facilitating various mobile learning activities. It is an app package including the following tool kits: KWL (a self-reflection tool which structures students reflection activities into three sections: (1) what I know, (2) what I want to learn, (3) what I have learnt), Notepad (a note taking tool), Blurb (a questioning tool), Sketchbook (an image editing tool), MapIT (a concept mapping tool), and Recorder (a voice recording tool). Involving these functional mobile tools, the student version serves for students’ participating in different mobile activities. Below is the interface of MyDesk student version (Fig. 1) and an exemplar of student doing a KWL activity (Fig. 2). Teacher version offers an authoring tool for the teacher to design the mobile activities and assign the tasks which incorporated the use of mobile tools above. Meanwhile, teachers can review and comment students’ posts in any forms.

For example, students did an experiment on the property of materials (hardness, softness, strength, waterproof, etc.) in the classroom and recorded the phenomena (i.e., using Notepad tool). Group work permitted students to work in collaboration in taking notes and discussing the phenomena (i.e., using Notepad tool and Recorder tool). Individual task was designed for students to input reflections on their prior knowledge (i.e., using KWL tool). Students also participated in the out-of-classroom and inquiry activities in field trips with the use of mobile tools. When they went back to the classroom, teachers reviewed their work and commented or graded the work (i.e., learning artifacts, reflections, and discussion) through the MyDesk learning management system as the follow-up of students’ activities in the informal context. This helps students to further elaborate the understanding and better connect the learning in the informal contexts and formal contexts. For more information about M5ESC, please refer to Looi et al. (2014a) and Looi, et al. (2014b). In brief, M5ESC provides students with various opportunities to engage in different types of activities and to build knowledge on the basis of inquiry in formal and informal learning contexts.

In addition, these tools are flexibly integrated with the learning activities under the consideration of the students’ cognitive levels based on Starkey’s Digital Learning Age Matrix (Starky 2011). Level 1 (doing) activities include the use of Notepad or/and Recorder for collecting data and writing notes in field trips. KWL allows self-reflection on the connections between knowledge; hence, it can be integrated into high cognitive levels of activities (i.e., level 2—thinking about connections, level 3—thinking about concepts, and level 4—critiquing and evaluating). The MapIT can be either integrated into levels 2, 3, and 4. As an image editing tool, Sketchbook is used to promote the students’ ability to connect knowledge with daily experiences and develop higher levels of conceptual understanding (i.e., level 5—creating knowledge and level 6—sharing knowledge) through peer assessment of artifacts. Blurb is generally used to improve students’ thinking and reasoning about the concepts through posing questions, which is appropriate for designing level 2 and level 3 activities.

With the above mentioned features, the M5ESC aims to promote students’ conceptual understanding in science and develop crucial learning skills, such as self-reflection thinking skills, collaborative learning skills, and self-directed learning skills.

### Participants

The participants were 310 students from eight grade 3 classes of a pilot school in Singapore. These students were divided into eight classes (3A, 3B, 3C, 3D, 3E, 3F, 3G, and 3H). The eight classes were further divided into three levels of ability by the school, named as HA (high achievement classes A, B, C), MA (mixed achievement classes E, F), and LA (low achievement classes D, H) based on students’ prior achievements at the P1/P2 level. Five experienced teachers were responsible for teaching P3 science. As a pioneer future school incorporating ICT in education in Singapore, the principal and teachers placed great emphasis on the implementation of the M5ESC innovation in the school, and they demonstrated their enthusiasm and passion toward the M5ESC development and implementation. They and their students had accepted the mobile learning as the routine in science learning both in and out of the classroom. It was common that when the teacher raised a question about a new concept, the students would bring out smartphones to search the online information; when a student was doing an experiment, his or her partner took the pictures of the phenomena as the evidence; if the teacher asked students to do reflection, the students would prefer to write reflection in KWL. A regular meeting was conducted on a biweekly basis for the teachers and researchers to share ideas on the lesson design and lesson enactment and elaboration. Thus, the M5ESC was iteratively improved by continuous cycles of teachers’ implementations and of interactions between teachers and researchers.

### Topics of M5ESC in P3 science

Table 1 shows the topics and the number of mobile learning activities in P3 science. There were 36 activities, with 8 KWL, 17 Sketchbook, 5 MapIT, 3 Blurb, 2 Notepad, and 1 Recorder activities designed in the curriculum of all the topics. The number of mobile activities in each topic varied considering the content knowledge and the learning objectives stated in the national science syllabus. Particularly, with emphasis on developing students’ self-reflection skills and self-directed learning skills, KWL learning activities were designed for each topic. The curriculum proposed learning science through daily life experience, so Sketchbook activities which enabled students to connect links between daily life knowledge and knowledge learnt in the classroom were frequently designed.

**Table 1:** The number of MyDesk learning activities in the P3 topics

Topic	MapIT	Recorder	Sketchbook	KWL	Blurb	Notepad
Animals	0	0	4	1	0	0
Plants	1	0	3	1	0	1
Fungi and bacteria	1	0	5	1	2	0
Materials	0	0	1	1	0	0
System	0	0	0	1	0	0
System plants	0	0	2	1	1	0
Body system	1	0	0	1	0	0
Digestive system	1	1	2	1	0	1
Total	5	1	17	8	3	2

### Data sources and data analysis

In the M5ESC project, research data was collected from 2009 to 2014. In this study, research data collected in 2013 school year was used and analyzed for research purpose. The data sources included observation sheets and field notes used in classroom observation, transcripts of teacher and students’ discourses, and students’ learning artifacts generated in the MyDesk system in and out of the classroom, as well as the pre- and post-tests before and after the implementation. As the data collection followed the school schedule, each lesson enacted in the intervention year of project was observed, and the data was collected and analyzed. Therefore, a huge database of project was accumulated from the year 2009 to year 2014.

In this study, a part of project data was utilized and analyzed for the research purpose. In data analysis, first, students’ activity participation in different mobile learning activities were statistically analyzed, which was used to indicate students’ engagement in the mobile learning activities. Thus, the number of completed mobile learning activity at each topic was first calculated, and the completion rate (the percentage of the completed activities) of each kind of activity was then generated. Quality of the learning artifacts generated in the higher participation activities (i.e., KWL, MapIT, and Sketchbook) was further identified for exploring students understanding levels through engaging the mobile learning activities. The identification of the quality levels were based on the content analysis of the learning artifacts following criteria: the KWL which received students’ reasonable reflections in the three sections of “what I know,” “what I want to know,” and “what I have learnt”; the concept map in MapIT which described the major components of the concepts and the relations among the components; and the images and relevant notes in Sketchbook that suggested students’ thinking and observation of science phenomena. These were identified as the high-quality learning artifacts. A high completion rate with students’ high-quality learning artifacts suggested their engagement in the mobile learning activities. Moreover, as we expected, there should be differences among tools use in different topics and classes, and students’ differences were analyzed and compared accordingly. Therefore, the class performance in responding to mobile activities were compared and discussed for exposing the relationship between the class level and the activity participation. Quantitatively, correlation relationship between teacher feedback and student activity performances was analyzed to find out the factors contributing students’ difference in participating the mobile activities. The descriptive analysis and paired samples *t* test were conducted to explore whether these differences were statistically significant.

## Results and discussion

### Students’ general performance in mobile activities

#### Activity participation

At this section, we focused on highlighting students’ tendency on the participation of the different mobile activities that which tools they preferred to use in describing their understanding. In 2013, MyDesk activities were designed and implemented in the whole cohort of P3 science lessons. The number of the activities supported by the mentioned tools scattered in the topics was different as we listed above. Table 2 is the descriptive analysis of students’ participation rates in each kind of mobile activity. For example, the mean participation rate of Recorder activities is 6.5 %, means among all participated students (*n* = 310), only 6.5 % (*n* = 20) used the voice recorder tool for recording their thinking or observation in doing the assigned Recorder activities.

**Table 2:** Descriptive statistics of MyDesk learning activities

MyDesk activities	Minimum	Maximum	Mean	Std. deviation
Recorder	.00	.52	.0650	.18385
Blurb	.00	.44	.0581	.14372
KWL	.00	1.00	.5239	.33754
MapIT	.00	.60	.0550	.15091
Sketchbook	.00	.98	.3631	.31999
Notepad	.00	.48	.0588	.12154

Overall, Table 2 shows that the participation rate of KWL activities was the highest among all the activities designed (average students participation rate was 52.39 %, mean almost 162 students among 310 students responded to KWL activities). This suggested that KWL was the most prominent mobile activity in all the classes. Specifically, in some of the classes, all the students had finished the KWL assignments, with the students’ participation rate being 100 %. Sketchbook activities enjoyed comparatively popularity with an average students’ participation rate of 36.31 %. However, students’ participation in the MapIT (5.5 %) and Blurb (5.81 %) was more limited. Their average completion rates were very low. We infer that students were mostly not familiar with these tools and they might have some difficulties in doing these activities outsides. Another key reason was that the teachers devoted most of time to the use of KWL and Sketchbook, and students had more opportunities to practice KWL and Sketchbook activities. Students were seldom engaged in activities on the Recorder and Notepad to support their learning. These all suggested that although mobile activities were designed for each topic, the participation rates of students in these activities differed.

#### Significance of activity participation

Paired samples *t* test further confirmed the discrepancies in the completion rate of different types of activities. According to the statistics, KWL was the most popular. The participation rate of KWL was significantly higher than that of Recorder (*t* = 10.032, *p* < .000), Blurb (*t* = 12.666, *p* < .005), MapIT (*t* = 13.646, *p* < .005), and Notepad (*t* = 13.056, *p* < .005). Sketchbook, whose participation rate was the second highest, was significantly higher than that of Blurb (*t* = 7.408, *p* < .005), Notepad (*t* = 7.134, *p* < .005), and MapIT (*t* = 11.092, *p* < .005). The test results also showed that there were significant differences between the use of KWL and Recorder, KWL and Blurb, Bulb and Sketchbook, KWL and Notepad, and MapIT and Notepad, as the Sig. (two-tailed) were 0.000. Therefore, the participation differences among the use of tools were significant.

#### Quality of learning artifacts

Among the completed activities, there were a considerable rate of good learning artifacts which suggested students thinking and understanding levels when doing the activities. Table 3 is the exemplar of satisfied KWL reflections. The reflections posted at the beginning, during, and the after learning of the fungi suggested a process of student’s conceptual understanding and the learning gains. This kind of reflections conveyed the valuable information of students thinking in and out of the classroom. Among all the reflections responded to KWL, we found 70 % of them were the satisfied KWL reflections, and the rest of them were reluctant to respond to all the sections of the KWL activities. Generally, the KWL activity engaged most of students in generating the reflections upon conceptual understanding and provided the teacher more comprehensive information of what his students’ thinking about their learning.

**Table 3:** Students’ reflections in the topic of fungi

What I know	What I want to	What I have learnt
• Spores are living things. • Colorful fungi (some) are deadly.	• How are fungi “created”? • How can fungi help the environment? • How does fungi grow on animals/humans? • Will poisonous fungi help the environment? • Will spores die? • Why are there fungi in this world?	• Fungi can be eaten, can be poisonous, and DEADLY. • Fungi can reproduce at least thousands of spores at a time. • Fungi can grow on humans and animals. • Spores are living things. • Fungi come in different shapes and colors. • Spores are all around us.

**Fig. 3:**
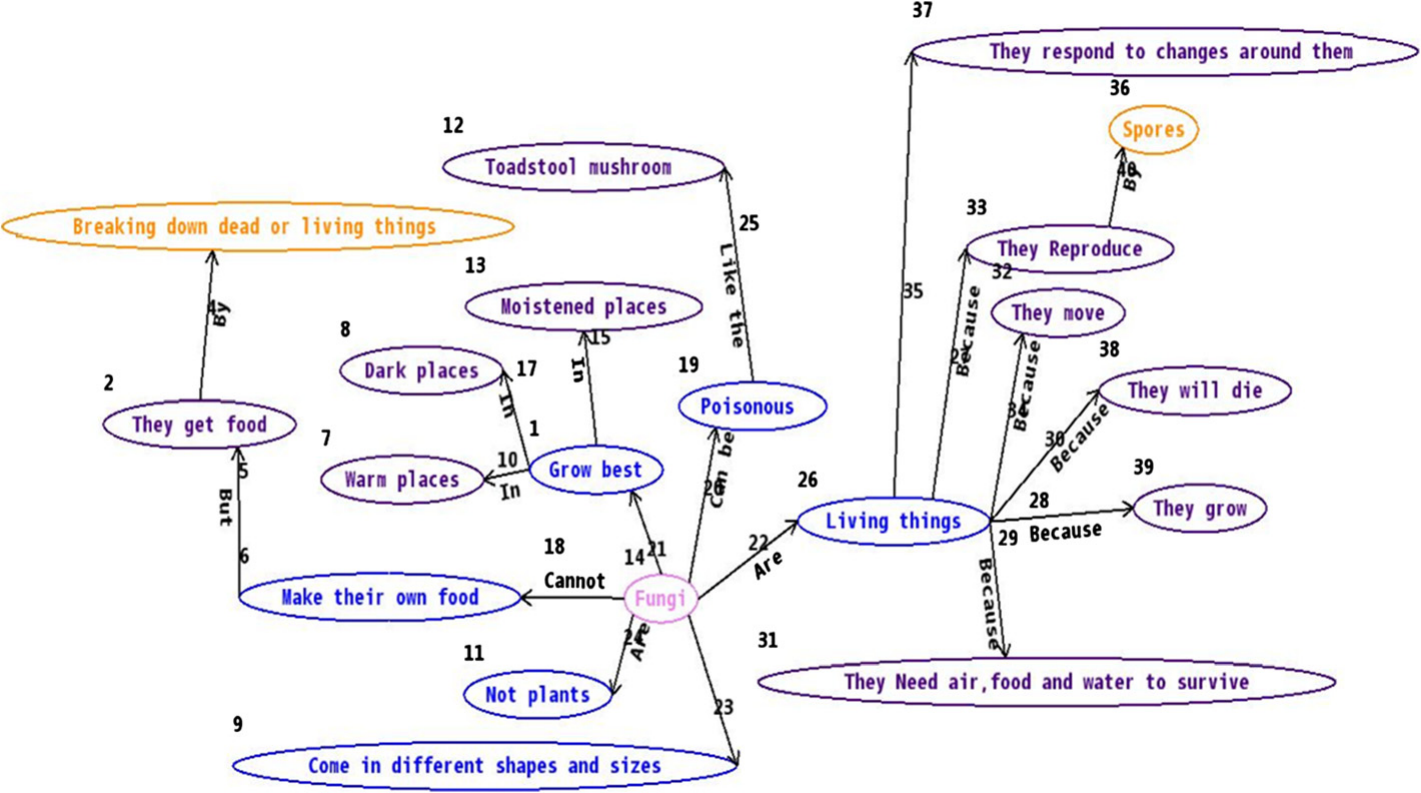
The concept map of fungi

**Fig. 4:**
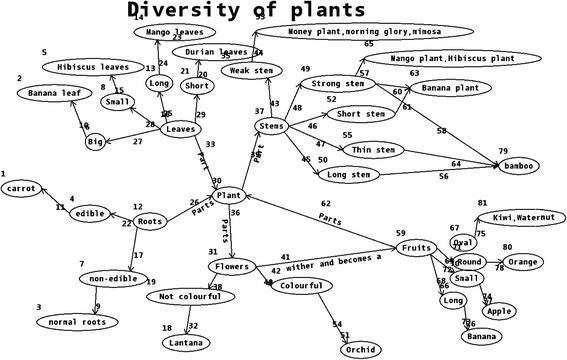
The concept map of plants

Although students constructed limited concept maps in MapIT activities, we found that a small group of students generated the high-quality concept maps. These concept maps reflected that some students had already understand the concept system in systemic way. It was found that among the existing concept maps, 80 % of them were the satisfied learning artifacts. Below are the typical examples. Figure 3 shows students’ understanding of the characteristics of fungi. Figure 4 shows students’ understanding of the plant’s parts and classification. These concept maps presented the value of MapIT for promoting students’ deep understanding of the concepts.

**Fig. 5:**
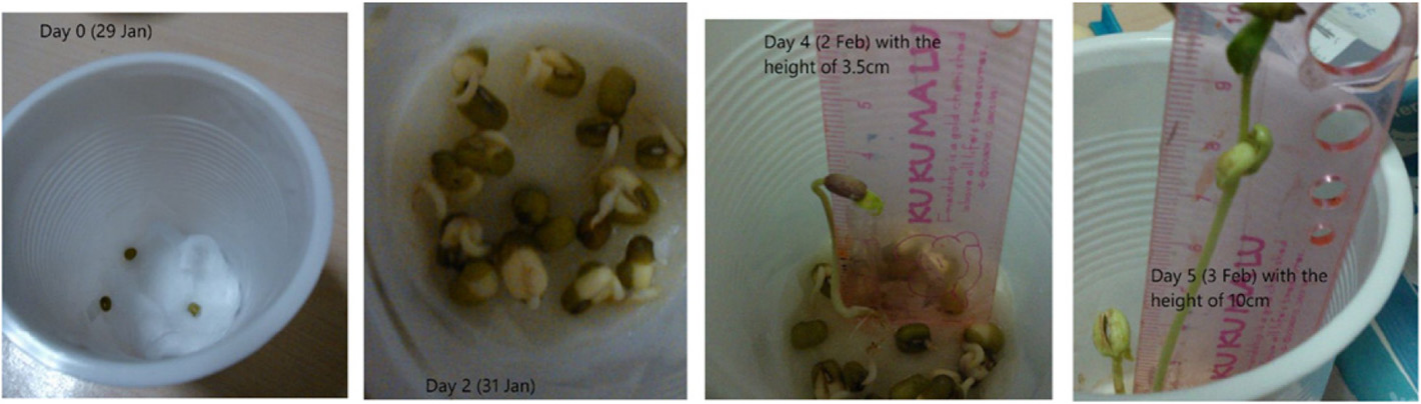
The growth of beans

**Fig. 6:**
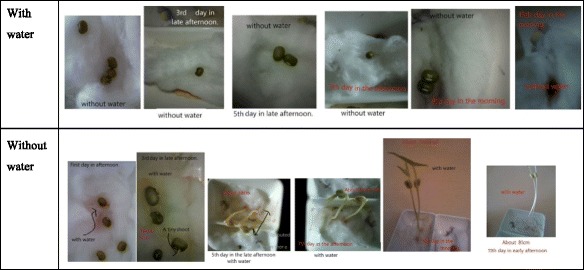
The growth of beans with and without water

The Sketchbook tool provided students with more opportunities of interacting with the outsides. In M5ESC, the most frequent activities were the Sketchbook activity. For example, in the topic of plants, the teacher assigned a task for observing the beans’ growth. The students were required to plant bean seeds, do observation, and take the pics of bean’s growth with explanations using Sketchbook tool. Among the existing Sketchbook learning artifacts, 90 % of students were engaged in the Sketchbook activities. The images of science phenomena taken became reliable evidence for suggesting students’ interests and motivation in doing these activities. More importantly, their thinking and reasoning process were better exposed by these learning artifacts. Below are the two exemplars of learning artifacts created by Sketchbook. Figure 5 depicts a process of bean growth. The student recorded the growth phenomena of beans through comparing the height of beans. The experiment also suggested that the student knew the measurement of the variable and the method of collecting data in a science experiment. Figure 6 is another student’s work at Sketchbook activity. This student applied the control variable method for conducting the science experiment at home. More evidence were collected in his home activity. Thus, with the use of Sketchbook, students engaged more in the out-of-classroom activities. And they could conduct more hands-on activities or home activities for relating the knowledge learnt in classroom and out-of-classroom contexts.

### Students’ responses to the topics

**Fig. 7:**
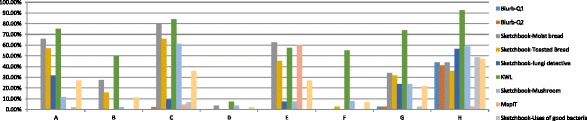
Participation rates of different classes for the topic of fungi and bacteria

Moreover, class responses to the mobile activities in different topics were also different. Take the topics of fungi and bacteria and the digestive system as typical examples. Figure 7 shows that in the topic of fungi and bacteria, generally, the class average participation rate of the MyDesk activities was not very satisfying even though all classes to some extent attempted the activities. The highest participation rate was achieved by class H (47.01 %) which was a LA class. The second highest was attained by class C (34.85 %), which was a HA class. Classes E and G had completed more than 20 % of the activities. The class performed worst was class D, with a participation rate of only 1.65 %. The participation rate of different types of activities differed sharply. The KWL activity was completed most thoroughly, with the highest participation rate of 92.31 % achieved by class H and an average participation rate of more than 50 % across classes. The second popular was the four Sketchbook activities designed. All the classes had attempted this type of activity. Blurb and MapIT activities were rarely completed. There were several classes that left these activities untouched (e.g., classes B, E, and F).

**Fig. 8:**
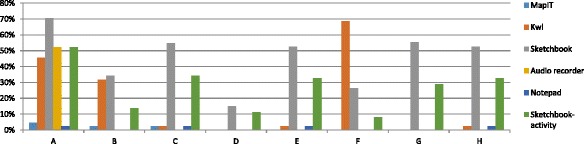
Participation rates of different classes for the topic of the digestive system

In the topic of the digestive system (Fig. 8), the participation rate, in general, was not very satisfying. Only one class out of seven had attained a participation rate of more than 20 %. The best performance was by class A with a participation rate of 37.88 %. Class D had the lowest participation rate of 4.32 %. Among different types of activities designed, MapIT, Recorder, and Notepad activities were hardly attempted in most classes. There was more participation in Sketchbook and KWL activities. In the KWL activity, the differences among classes were very obvious. The highest participation rate was 68.42 % by class F, yet there were classes (classes D and G) who did not finish the activity at all. Thus, the response of the same class in one topic was not consistent with the responses to other topics. These suggested that even in the same class, different participation rates of the mobile activities were generated. We infer that influenced by teachers’ lesson enactment and students’ ability levels, class differences in the participation level of mobile activities occurred.

### Class levels and responses

**Fig. 9:**
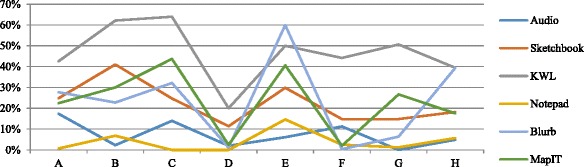
Class participation in the mobile activities

Besides the difference of responses to mobile activities according to the topics, there were also differences in different class levels as we mentioned above. Figure 9 shows that among all the classes, HA classes A, B, and C generally completed more mobile activities than the MA classes E, F, and G, while LA classes D and H generated comparatively less KWL reflections. For HA classes, class C contributed to more Sketchbook, Blurb, and MapIT activities. For the MA classes, class E performed the most in all activities, while F completed the least MyDesk activities. LA class H performed well, especially in the Sketchbook, MapIT, and KWL activities, providing a high completion rate on average. Thus, although HA completed more mobile activities in general, there were negative responses for Notepad and MapIT activities. MA class E performed better than the HA class A. This suggests that class ability may not be the only key factor on students’ participation of the mobile activities, as other factors may further affect their participation rates.

### Teacher feedback

In P3 science, the five teachers were teacher (A), teacher (B, F), teacher (C, E), teacher (D, G), and teacher (H). As MyDesk allows teachers to grade and comment students’ learning artifacts, we analyzed teacher feedback and found that teacher difference existed in response to students’ mobile activities. Rapid and frequent feedback were generated by some teachers, while some teachers ignored most of students’ work. For example, the class H performed the best when they learnt the topic of fungi and bacteria, and meanwhile, we noted that the teacher (H) commented all learning artifacts in this topic. While in the digestive system, the teacher (H) only provided feedback to Sketchbook activities, which resulted in the higher participation of the sketchbook. In 2013, students’ MyDesk activities involving KWL, Blurb, and Sketchbook received more teachers’ feedback compared to other activities. Table 4 shows that strong correlation was detected between Blurb’s and Sketchbook’s engagement and teacher feedback. Significant correlation was detected between KWL engagement and teacher feedback. This reveals that teacher feedback was one of the key factors that affected students’ participation of the mobile activities. This may help us explain why some MA classes complete more mobile activities than some HA classes as mentioned above, and the LA class H had better performance.

**Table 4:** Correlation of teacher feedback and students’ response to the mobile activities

Feedback to the activities	Correlation	
KWL 2013P3-feedback	Pearson correlation	.276*
	Sig. (two-tailed)	.039
Blurb 2013P3-feedback	Pearson correlation	.997**
	Sig. (two-tailed)	.000
Sketchbook 2013P3-feedback	Pearson correlation	.457**
	Sig. (two-tailed)	.000

*Correlation is significant at the 0.05 level (two-tailed); **correlation is significant at the 0.01 level (two-tailed)

On the other side, teacher performed differently in responding to students’ mobile learning activities, which lead to the difference of class performance. Among these teachers, teachers who taught classes C (feedback for 34.85 % of activities on average), G (feedback for 21.64 % of activities on average), and H (feedback for 47.01 % of activities on average) performed most actively in providing feedback to the students. Being different from other teachers, the teacher who taught class H provided feedback for each mobile activity, leading to the equally high participation in the mobile activities in class H. For example, in the topic of fungi and bacteria, the rate of teacher feedback to the activities was in general lamentable, except for the teacher who taught class H achieved a satisfying 86.71 % average feedback. Except for the MapIT activity in which the teacher did not provide feedback, the other activities all received good amount of feedback. This may be the reason of lower participation rate of MapIT activities. The feedback to Sketchbook activities was all good at 100 %. The teacher managed to provide feedback to 86.36 % of class B’s work in KWL, but for other activities, the feedback rate was 0. Therefore, these findings suggest that teacher difference has been the major factor for affecting students’ motivations of participating the mobile activities.

## Conclusions

To address the problems on the research of mobile learning, which focus more on the discussion of learning outcomes rather than exposing the learning process, the study attempts to unfold the missing aspects of students learning in the out-of-classroom activities. The study presents an exploration of students’ engagement in the mobile activities in the context of a science curriculum implementation supported by mobile technologies. The paper provides the general information of the curriculum, especially for the design features of mobile learning activities, which were shaped on the basis of long-term curriculum development and implementation. With an aim of detecting students learning process in the out-of-classroom settings, the study borrowed part of project data (i.e., learning artifacts created by mobile tools). The findings on the difference of students’ activity participation were exposed and discussed. Meanwhile, some high-quality learning artifacts generated by different mobile tools were illustrated and discussed for confirming the effectiveness of well-designed mobile learning activities (Chu 2014). The findings also enable us to answer the research question 1: How to capture students’ activity performance in the informal contexts? Regarding research question 2: What are the differences of the participation rates when students doing various mobile leaning activities? We found that students responded differently to different mobile learning activities. And the students with different levels were also performed differently in the participating in these activities. The findings of the correlation between teacher feedback and students’ activity performance well answer the research question 3. In sum, the results showed that although various mobile activities designed for learning science concepts, the students’ participation in these mobile activities were varied because of students’ ability, topics, class levels, and teacher feedback. The points which were limited discussed in relevant studies. Moreover, the investigation of students’ performance in mobile activities would be the starting point of further study of students’ behavior in thinking and reasoning process.

Regarding the reasons of causing the differences in participation in the mobile activities among students, we summarized the following aspects based on the data analysis and discussion above:

Relevant study showed mobile technological design that may impact the mobile use for learning within a mobile learning environment. This evidence strengthens the need for technology developers and learning content designers to address mobile device usage and type as essential design considerations for mobile learning needs (Reychav et al. 2015). Therefore, for mobile technology-supported curriculum design, more instructions should be provided for promoting students participations in the mobile learning activities. The instructions, such as the instructional scripts will guide students to better use the mobile tools. This suggestion is based on Falk and Balling (1982)’s study. They found that without orientation and preparation, students were more likely to concentrate on non-relevant aspects of the surroundings, rather than those relevant to the learning intended. Moreover, the appropriate use of the mobile tools is proposed in and out of the classroom for addressing the educational value of different mobile tools. The way will solve the problem that students’ preference of some frequent used mobile tools and ignorance of the tools rarely used in curriculum.

For curriculum implementation, teacher feedback should be scattered equally in response to students’ mobile learning artifacts. This will improve students’ participation in the activities significantly. The teachers are also encouraged to conduct more follow-up/post-activities related to the outsides tasks in the classroom for assessing students’ performance, highlighting misunderstanding, clarifying remaining questions or reinforcing concepts, which has been frequently discussed and in the relevant studies (DeWitt and Osborne 2007; Rickinson et al. 2004). These activities are the key connection between students’ learning in and out of the classroom and the best way for the teacher to detect students learning and elaborate students’ understanding.

Regarding class levels, the mobile activities should be designed and instructed differently. For the LA classes, more instructions and progress monitor should be provided. And the teachers should devote more efforts to these for engaging more low ability students in the activities. For the HA class, the outside activities are proposed to design for encouraging students to do personal inquiry of the science phenomena and elaborate their understanding and develop inquiry skills based on the outside inquiry activities.

We also recognized the importance for developing teacher competencies on teaching outside learning activities to fit into the standard curriculum. In current teacher training programs, this area has been somewhat neglected. The training should focus on developing teaching strategies, the pre-, during, and post-activity implementation. As Phillips (2007) found that recent teacher professional development programs tend to blend the elements of the less structured setting of learning in the informal science institutions with the more structured requirements and goals of the K-12 educational system, but the need to determine how learning in the informal context can best support students and teachers in terms of actual curriculum and materials used within the classroom.

With the development of the mobile learning in both technology and pedagogy, calling for in-depth investigation on the value of mobile learning is necessary. Based on our research experience and the literature review of the recent relevant studies, future research should focus more on the exploration of the enactment of mobile technology-supported lessons/curriculum, with the aim of studying the factors that affect the teacher and students’ behavior. The results will inform the design and enactment of lessons supported by the mobile technology.
